# Application of Spatial Offset Raman Spectroscopy (SORS) and Machine Learning for Sugar Syrup Adulteration Detection in UK Honey

**DOI:** 10.3390/foods13152425

**Published:** 2024-07-31

**Authors:** Mennatullah Shehata, Sophie Dodd, Sara Mosca, Pavel Matousek, Bhavna Parmar, Zoltan Kevei, Maria Anastasiadi

**Affiliations:** 1 Centre for Soil, Agrifood and Biosciences, Cranfield University, College Road, Cranfield, Bedford MK43 0AL, UK; 2 Central Laser Facility, Research Complex at Harwell, STFC Rutherford Appleton Laboratory, UKRI Harwell Campus, Didcot OX11 0QX, UK; 3 Food Standards Agency, Clive House, 70 Petty France, Westminster, London SW1H 9EX, UK

**Keywords:** honey, SORS, random forest, classification, regression

## Abstract

Honey authentication is a complex process which traditionally requires costly and time-consuming analytical techniques not readily available to the producers. This study aimed to develop non-invasive sensor methods coupled with a multivariate data analysis to detect the type and percentage of exogenous sugar adulteration in UK honeys. Through-container spatial offset Raman spectroscopy (SORS) was employed on 17 different types of natural honeys produced in the UK over a season. These samples were then spiked with rice and sugar beet syrups at the levels of 10%, 20%, 30%, and 50% *w*/*w*. The data acquired were used to construct prediction models for 14 types of honey with similar Raman fingerprints using different algorithms, namely PLS-DA, XGBoost, and Random Forest, with the aim to detect the level of adulteration per type of sugar syrup. The best-performing algorithm for classification was Random Forest, with only 1% of the pure honeys misclassified as adulterated and <3.5% of adulterated honey samples misclassified as pure. Random Forest was further employed to create a classification model which successfully classified samples according to the type of adulterant (rice or sugar beet) and the adulteration level. In addition, SORS spectra were collected from 27 samples of heather honey (24 *Calluna vulgaris* and 3 *Erica cinerea*) produced in the UK and corresponding subsamples spiked with high fructose sugar cane syrup, and an exploratory data analysis with PCA and a classification with Random Forest were performed, both showing clear separation between the pure and adulterated samples at medium (40%) and high (60%) adulteration levels and a 90% success at low adulteration levels (20%). The results of this study demonstrate the potential of SORS in combination with machine learning to be applied for the authentication of honey samples and the detection of exogenous sugars in the form of sugar syrups. A major advantage of the SORS technique is that it is a rapid, non-invasive method deployable in the field with potential application at all stages of the supply chain.

## 1. Introduction

Honey is a sweet substance produced naturally by honeybees and is a popular food product consumed globally for its taste, nutritional value, and perceived health benefits [1]. UK domestic production is far from self-sufficient, and recently, the UK was listed as one of the top four countries worldwide importing honey [2] and the second-largest importer in Europe, with 51,912 tonnes of honey imported in 2022, valued at EUR 122 million [3]. However, the total honey imports in Europe are expected to show little growth in the coming years, with consumer concerns around the purity and authenticity of honey cited as one of the main reasons [3]. These concerns were further exacerbated by the recent European Commission’s Joint Research Centre (JRC) report published in 2023, which identified that 46% of the 147 samples tested were suspected of being adulterated [4]. Current analytical methods are inadequate for definitively detecting adulteration, requiring additional research and traceability information collection.

A recent review commissioned by the UK government highlighted the need for research into new standard analytical tests for honey authentication, partly due to the high-level interpretation needed in existing approaches [5,6]. The same report identified some of the possible malpractices associated with honey, including direct adulteration with sugar syrups and mislabeling of origin.

Sugar adulteration of honey involves diluting pure honey with cheaper sugar syrups, which not only results in inferior taste but also alters the nutritional properties and chemical composition [7]. The standard method for exogenous sugar detection in honey is based on a stable carbon isotope ratio analysis (SCIRA), which relies on identifying the presence of adulterant C4 plants by assessing the 13C/12C ratio [8]. However, SCIRA is not suitable for syrups derived from C3 plants such as rice and sugar beet, with the recent 2023 EU JRC technical report indicating that SCIRA methods (AOAC method 991.41) were not effective in detecting honeys suspicious of non-compliance, suggesting that sugar syrups from corn or sugar cane are no longer being used to adulterate honey for the European consumption [4]. An additional issue with this method is the presence of false positives and differences between testing labs reported by beekeepers [9], which ultimately means samples require additional and more costly analyses to prove authenticity; hence, it is evident that more robust and repeatable standard tests are needed.

Recently, spectroscopic methods including infrared (IR), Raman, fluorescence spectroscopy, and nuclear magnetic resonance (NMR) spectroscopy have been proposed as alternative methods for the rapid determination of the sugar adulteration of honey. These methods rely on mapping the spectral fingerprint of pure and adulterated honeys and applying multivariate data analysis techniques to build predictive classification and regression models able to identify adulterated samples. Notably, H-NMR was one of the methods employed during honey testing in the recent 2023 EU JRC technical report.

Raman spectroscopy has also been proposed as a suitable technique for honey authentication. It has been used as a rapid, non-destructive method for adulteration detection in honey using the spectral fingerprint of each component. Oroian et al. (2018) used Raman spectroscopy and PLS-DA to detect sugar adulterants in honey, such as fructose, glucose, malt must, etc. [10]. Raman spectroscopy has also been combined with machine and deep learning algorithms such as Convolutional Neural Networks (CNNs) to detect the type and amount of adulterant in honey [11,12] or LDA to discern the botanical origin of monofloral Italian honeys [13].

In this work, we employed spatially offset Raman spectroscopy (SORS), a truly non-invasive technique which has been explored for a wide range of applications, including medical diagnostic tests, through-container detection of explosives, and non-destructive analysis of pharmaceutical products, among others [14,15,16]. SORS has also shown promising results for food authentication applications, including through-container detection of counterfeit alcohol [17] and authentication of dairy products such as butter and cheese [18,19]. SORS offers several advantages in analytical applications. It is non-invasive, allowing measurements of samples in situ [20] and analysis through barriers or packaging and providing subsurface information without the need for sample preparation or disturbance [21]. Moreover, SORS results in a higher sensitivity compared with conventional Raman spectroscopy by suppressing interfering signals emanating from the jar’s glass wall [22]. These properties make SORS a rapid portable screening method, which is particularly important for traceability purposes along the food supply chain [19].

This study aims to develop a novel methodology using SORS combined with machine learning to authenticate UK honeys and detect sugar syrups from various plant sources, including rice, sugar beet, and sugar cane.

## 2. Materials and Methods

### 2.1. Chemicals

Methanol (LC-MS grade) and acetonitrile (≥ 99.9%) were obtained from Fisher Scientific, UK. D-(−)-fructose (> 99%), D-(+)-glucose (> 99.5%), sucrose ≥ 99.5%), and maltose (> 95%) were sourced from Sigma Aldrich, Gillingham, UK.

### 2.2. Sample Collection and Preparation

Honey samples from the UK were collected in different years. In Year 1 (2019), different heather honeys were sampled from beekeepers and small producers across the UK (*n* = 27) with the majority of samples produced in Scotland, Wales, and the North of England. The floral origin of the honeys characterised as ling heather by the producers (*n* = 24) was further tested by Minerva Scientific Ltd. (Derby, UK) to determine the percentage of ling heather pollen (*Calluna vulgaris*) through microscopy. Three honeys which were characterised mainly as bell heather (*Erica cinerea*) by the producers were not tested for pollen. In Year 2 (2023), different floral types of UK honey (*n* = 17) were collected directly from UK beekeepers, with the aim to represent all different seasons of honey collection in the UK. Table 1 and Table 2 contain more details regarding the honey samples used in this study. Figure S1 shows a picture of the 17 honeys collected in Year 2.

Commercially available sugar syrups derived from rice (*n* = 7), sugar beet (*n* = 4), and sugar cane (*n* = 1) were purchased from online retailers or grocery stores in an effort to obtain sugar syrups from known plant sources (Table 3, Figure S2). The pure honey samples were liquified at 45 °C for 60 min and stirred until homogenous. In Year 1, a subsample of 6 randomly selected heather honeys were spiked with partially inverted sugar cane syrup (golden syrup) at the 20%, 40%, and 60% adulteration levels to a total mass of 10 g (*w*/*w*) and further incubated at 45 °C for 60 min in a water bath, with constant agitation to obtain a homogenous mixture before being transferred to 7 mL Pico Glass vials with 16.7 mm diameter (Perker Elmin, Beaconsfield, UK) for SORS measurements. In Year 2, each honey was spiked with rice syrup (r01) and sugar beet syrup (b05) (Table 3) at the 10%, 20%, 30%, and 50% adulteration levels to a total mass of 10 g (*w*/*w*) and further incubated at 45 °C for 60 min, as previously described, and subsequently transferred to 7 mL Pico Glass vials with 16.7 mm diameter (Perker Elmin, Beaconsfield, UK) for SORS measurements.

Apart from the honey and syrup samples, a set of individual sugar standards were prepared consisting of 50, 25, and 12.5% aqueous solutions (*w*/*v*) of fructose, glucose, sucrose, and maltose. Two HPLC water samples were also used as “blanks”.

### 2.3. SORS Data Acquisition

The SORS measurements took place at The Central Laser Facility (CLF, Rutherford Appleton Laboratory, Didcot UK) using a custom-made setup optimised to perform Raman measurements in a conventional point-like spatial offset Raman spectroscopy (SORS) described in detail elsewhere [21,23]. Briefly, the excitation source consisted of an 830 nm wavelength with a maximum power of 400 mW output power focused on a ~0.5 mm diameter size spot on the sample surface. The Raman signal was collected from a spot with an ~1.5 mm diameter at different spatial displacements (i.e., ‘so’) from the excitation location (Year 1: so = 0 mm; so = 2.5 mm, Year 2: so = 0 mm; so = 4 mm). For each sample, three different locations were measured as shown in Figure S3. Each SORS spectrum was acquired for both 0 and 4 mm spatial offsets using, for the Year 1 dataset, 200 mW laser power, an acquisition time of 10 s, and 4 accumulations (i.e., total time 400 s) and, for Year 2, 35 mW laser power, an acquisition time of 1 s, and 10 accumulations (i.e., total time 100 s). The reason for using different experimental parameters in Year 1 and Year 2 was that the SORS system was recently upgraded with new optical components, including a charged-coupled device (CCD) detector that improved the illumination and signal collection performance, which allowed us to reduce the power and acquisition time without compromising the Raman spectra quality.

### 2.4. Data Pretreatment and Exploratory Data Analysis

All data analyses were conducted using the R environment (R version 4.3.2). The dataset from Year 1 consisted of 47 samples measured in random order (pure honeys = 27, spiked honeys = 18, sugar cane syrup = 1, water (blank) = 1, with all measurements taken in triplicate) and 5 random samples measured again at different timepoints to account for any batch effects. The Raman spectra consisted of 1024 data points corresponding to the Raman shift (cm^−1^) between 112 and 1934 cm^−1^. The dataset from Year 2 consisted of 179 samples (pure honeys = 17, spiked honeys = 136, rice syrups = 7, sugar beet syrups = 4, individual sugar solutions = 4 at 3 different concentrations (50, 25, 12.5%), blank (water) = 2, with all measurements taken in triplicate plus 3 samples repeated at different timepoints).

The wavenumbers at the peripheries were cut so that the spectral range was restricted from 337.8 to 1470.9 cm^−1^ for Year 1 and 585 cm^−1^ to 1550 cm^−1^ for Year 2, which contained the structural information-rich areas of the spectra.

A baseline correction was subsequently performed using a modified polynomial fitting method from the R package baseline (version 1.3-4) [24]. This method subtracts the fluorescence signal from the fluorophores within the honey or syrup matrix, which suppresses the Raman signal, helping to improve the performance and robustness of the statistical and machine learning models.

The matrix of the baseline-corrected spectra was smoothed using the Savitzky Golay method for smoothing (differentiation order = 0, polynomial order = 7, and window size = 31) and differentiation included in the R library prospectr (version 0.2.6). This method reduces a signal high-frequency noise by smoothing and reduces the low-frequency signal using differentiation [25].

Following baseline correction, the spectra were normalised using the standard normal variate (SNV) method from the prospectr package. This method normalises each row of the smoothed matrices (corresponding to a single SORS measurement) by subtracting each row by its mean and dividing it by its standard deviation. It effectively eliminates the constant offset and multiplicative differences between spectra.

**Figure 1:**
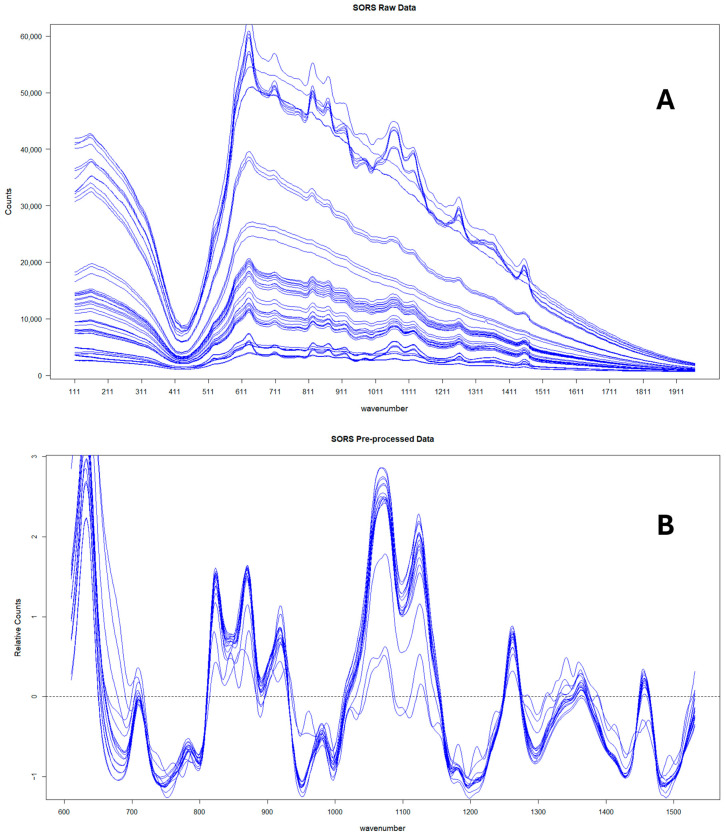
SORS spectra corresponding to the 17 pure honeys collected in Year 2 before (**A**) and after (**B**) preprocessing.

Finally, the mean of the three technical replicates was then taken for each sample. Figure 1 shows the average SORS spectra at spatial offset (4 mm) for the 17 pure honeys measured in Year 2 before and after preprocessing.

### 2.5. Exploratory Data Analysis Using Principal Component Analysis (PCA)

PCA is an unsupervised multivariate analysis which performs data reduction and denoising and can be used to visualise the natural clustering of the samples for exploratory data analysis purposes and quality control. PCA was performed separately for the Year 1 samples (pure and adulterated heather honey) and the Year 2 samples (17 types of pure and adulterated honey). Based on the PCA results for Year 2, a total of 14 honey types which showed similar spectral fingerprints were selected for developing machine learning classification and regression models.

### 2.6. Predictive Modelling Using Machine Learning

Predictive modelling was performed primarily on the SORS data obtained in Year 2 due to the availability of more extensive datasets representative of the UK origin honey production. A range of different classification and regression models were constructed based on the SORS spectra, with the aim of selecting the best-performing algorithm and assessing the potential of detecting the type and level of adulteration in UK honeys. The process was divided in the three stages described below:

(a) The first stage focused on creating separate models for rice and sugar beet adulteration detection. The process for selecting the training and test sets is displayed in Figure S4. The dataset for developing the rice syrup adulteration model consisted of the SORS spectra for the 14 pure honeys and the respective rice-spiked honeys at the 10%, 20%, 30%, and 50% adulteration levels (70 samples in total). Similarly, the dataset for sugar beet adulteration model consisted of the SORS spectra for the 14 pure honeys and the respective sugar beet-spiked honeys (70 samples in total). Next, each dataset was separated into training and test sets for the purpose of developing a classification model and testing its performance with an independent test set. For each syrup group, the training set was formed of 12 randomly selected pure honeys and their corresponding spiked samples (with rice or sugar beet syrup), while the test set consisted of the remaining two pure honeys and their corresponding spiked samples. A total of 91 possible combinations for separating the data into training and test sets were tried, and 91 models were subsequently built—per algorithm and adulteration type—in order to assess how small changes in the training and test data affect model performance.

The ML algorithms selected for performing the classification for rice and sugar beet syrup adulteration were the following: partial least squares–discriminant analysis (PLS-DA), Random Forest (RF), ordinal RF, and eXtreme Gradient Boosting (XGBoost). The resulting models for rice syrup adulteration assigned unknown samples to one of the following 5 classes: honey, 10% rice-spiked, 20% rice-spiked, 30% rice-spiked, and 50% rice-spiked. Similarly, the sugar-beet syrup adulteration models assigned samples to 5 classes consisting of pure honey and sugar beet syrup adulterated honeys.

The selection of algorithms was based on their strong track record for high prediction accuracy and suitability for high dimensional datasets.

PLS-DA is a popular algorithm widely used for classification tasks, particularly in complex datasets with high dimensionality and multicollinearity, such as spectroscopic data. It combines partial least squares regression with discriminant analysis, seeking to maximise the separation between classes while reducing the number of variables used for prediction [26]. In this study, PLS-DA was performed using the mixOmics library (version 6.24.0).

The RF algorithm is a robust ensemble learning method that operates by constructing multiple decision trees during training and outputs a pattern of classes (classification) or an average prediction of the individual trees (regression). In addition, the ordinal forest (OF) method allows ordinal regression for ordinal target values (categorical variables which can be ordered). Since the percentage of adulteration can be considered, an ordinal value OF was also tested in this work. By aggregating predictions from different trees, the RF algorithm minimises the risk of overfitting and improves prediction accuracy and generalisation to unseen data. Furthermore, by considering random subsets of features for each tree, it promotes diversity among individual trees, contributing to its efficiency in handling high-dimensional data and reducing variance [27]. All RF models were trained and optimised using the caret library in R (version 6.0-94). Prior to training the RF models, a feature extraction pretreatment step was also considered using RF to select the most important features; however, the performance results were considerably worse compared to the models built on the whole feature space, and therefore, this step was abandoned.

XGBoost is another ensemble technique based on decision trees which has gained popularity due to its effectiveness and efficiency. The algorithm works by iteratively building an ensemble of weak prediction models, typically decision trees, and adding them to the ensemble in a sequential manner. XGBoost utilises gradient descent optimisation techniques for model training and incorporates regularisation techniques such as shrinkage and pruning to prevent overfitting and improve generalisation performance [28]. The XGBoost models were trained using the xgboost library (version 1.7.7.1). An added benefit of using XGBoost is that the output for each prediction, consists of the respective probabilities of the sample belonging to each of the possible classes included in the model.

An additional advantage of all three selected algorithms is their ability to identify relevant features or predictors contributing to the classification or regression outcomes, enhancing the interpretability of the prediction models. This can be achieved by studying the variable importance graphs for each model.

(b) The second stage consisted of building a combined classification model for identifying both the type of adulterant (rice or sugar beet syrup) and the level of adulteration. The dataset for developing the combined adulteration model consisted of the SORS spectra for the 14 pure honeys and the respective rice and sugar beet spiked honeys at the 10%, 20%, 30%, and 50% adulteration levels (126 samples in total). Then, the dataset was separated into training and test sets (91 combinations) following the same approach described in stage 1, except this time, each test set included two pure honeys and the respective spiked honeys with both rice and sugar beet syrup. The algorithm employed for developing this model was RF (classification).

(c) The final stage consisted of building regression models for rice syrup and sugar beet syrup adulteration, with the aim to predict the percentage of adulteration as a continuous numerical value rather than a categorical value. The algorithm employed for regression purposes was RF (regression).

Finally, we used the SORS data obtained in Year 1 in combination with RF to build a binary classification model for predicting “pure” vs. “adulterated” heather samples and an ordinal regression model for categorising the samples into the “pure”, “low”, “medium”, and “high” adulteration levels.

### 2.7. Performance Metrics

For each of the classification models, the confusion matrix for each of the 91 data combinations was used to extract the model accuracy, i.e., the ratio of correct predictions over the total number of predictions and the total error = 1-accuracy. However, to gain more information on the ability of each model to differentiate between pure and adulterated honey, we introduced two more metrics, the “hard” and “soft” misclassifications. Among the “hard” misclassifications were the ratio of pure honey misclassified as adulterated and the ratio of adulterated honeys misclassified as pure. These are considered as the most serious types of misclassifications; thus, they were captured separately. In contrast “soft” misclassifications were considered less serious errors and represented the cases where an adulterated sample was classified as adulterated but under the wrong adulteration level. The soft misclassification ratio was also captured during this study.

The metric used for the regression models was the root mean square error (*RMSE*), as shown in Equation (1).

(1) $$RMSE=\sqrt{\frac{\sum_{i=1}^{n}{\left(\hat{y}-y\right)}^{2}}{n}}$$

where *n* = the number of samples in the test set;

$\hat{y}$ = the predicted value;

y = the actual value.

### 2.8. Sugar Analysis Using HPLC-ELSD

A sugar analysis was also undertaken during the project to identify and quantify the concentration of individual sugars present in the syrup samples. All samples were prepared and analysed according to the Harmonised Methods of International Honey Commission [29] with some modifications. A chromatographic analysis was performed using a mobile phase comprising acetonitrile–water (80:20, *v*/*v*) with a flow rate of 0.8 mL/min and a sample injection volume of 10 μL. The column and detector temperature were held at 35 °C during the whole run. Detection was performed using an evaporative light scattering detector (ELSD) connected to an Agilent 1200 infinity HPLC (Agilent Technologies, Stockport, UK) fitted with a prevail carbohydrate ES 5 mm size of 250 nm × 4.6 mm diameter and a guard column of the same type. Fructose, glucose, sucrose, and maltose were quantified using external calibration curves of commercial standards.

## 3. Results

### 3.1. PCA Results for Rice and Sugar Beet Adulterated Honey Samples

**Figure 2:**
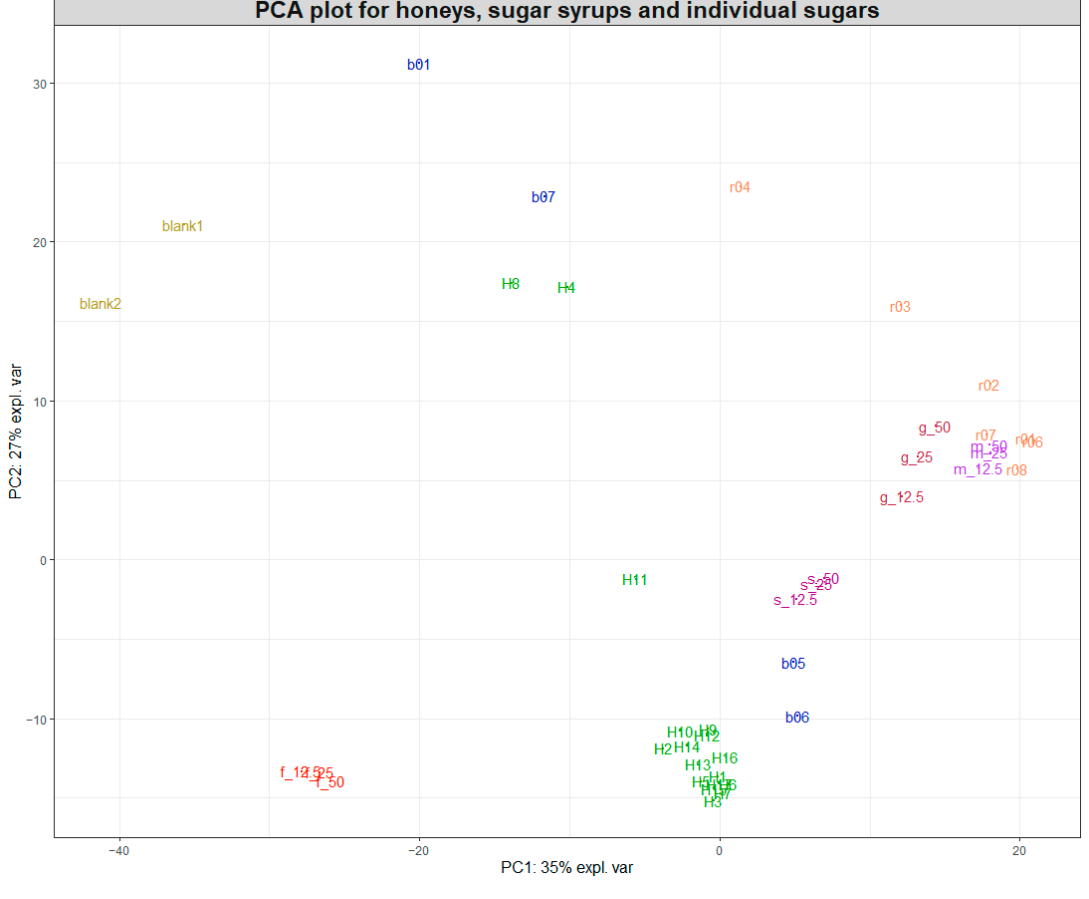
PCA plot of the SORS spectra acquired for honeys, sugar syrups, and sugar aqueous solutions. Pure honeys (H1–H17) are coloured in green, sugar beet syrups (b01 and b05–b07) in blue, and rice syrups (r01–r04 and r06–r08) in orange. The two blanks are water, while the individual sugars fructose (f), glucose (g), sucrose (s), and maltose (m) are 50, 25, and 12.5% *w*/*v* aqueous solutions.

Following the initial data pretreatment and normalisation (Figure 1), PCA was employed to visualise the clustering of samples for each of the two datasets acquired during the study. Figure 2 shows a PCA plot containing the 17 pure honey samples, the sugar beet and rice syrups, the individual sugar standards (50, 25, and 12.5% *w*/*v*) and the water samples (blanks). By observing the sample clustering, it becomes obvious that the majority of the pure honeys formed a tight cluster on the bottom left side of the PCA score plot, indicating similar biochemical profiles. The 14 honeys that clustered close together were woodland, sycamore, phacelia, Himalayan balsam, spring set, borage, meadowfoam, sea lavender, echium, field and forest, hedgerow, English blossom, apple blossom, and wildflower. Buckwheat (H8) and ivy honey (H4) were positioned away from the main cluster towards the upper middle area of the PCA plot, while heather honey (H11) was positioned in between. Figure S1 shows the colour differences among the different types of honey, with the ivy (H4) and buckwheat (H8) having distinctly dark amber colours.

Most rice syrups formed a cluster at the left top side of the PCA plot, with the exception of rice syrup r03 and r04, which were the darkest rice syrups, as shown in Figure S1.

Interestingly, maltose and glucose also clustered closely to the rice syrups, which indicated that they were the dominant sugars in rice syrups. On the contrary, two of the sugar beet syrups (b05 and b06) were positioned relatively close to the cluster formed by the 14 pure honeys, together with the sucrose aqueous solutions, while the other two sugar beet syrups were spread across the top left side of the PCA plot. Sugar beet syrups b05 and b06 were marketed as high fructose golden syrups, which might explain their proximity to the 14 honeys. On the other hand, b01 was marketed as molasses and b07 was a blend of a sugar beet and sugar cane syrup. Both of these syrups had a distinct dark amber-brown colour and produced a high-intensity SORS signal and strong fluorescence interference similar to the signal from ivy and buckwheat honeys, which were also dark amber honeys, as demonstrated in Figure S1.

The sugar composition of the sugar syrups and pure honeys was further confirmed by HPLC-ELSD, and the results are displayed in Table S2. The sugar composition results show that the dominant sugars in all rice syrups were maltose and glucose, with concentrations ranging from 28.57 to 42.59 g/100 g^−1^ for maltose and from 9.54 to 28.11 g/100 g^−1^ for glucose. Rice syrup r01, which was used for spiking the honey samples, contained 31.90 g/100 g^−1^ maltose and 22.49 g/100 g^−1^ glucose. Rice syrups r03 and r04 exhibited the lowest glucose concentrations among the rice syrups, at 17.34 and 9.54 g100 g^−1^, respectively, while r04 also had the highest maltose concentration, indicating a lower degree of maltose conversion to glucose in this syrup. Figure S5A, depicting the processed Raman spectra for all the rice syrups along with maltose and glucose solutions (50%*w*/*v*), also shows significant variations in the Raman profiles of r03 and r04. However, by looking at the biplot in Figure S5B, showing the 150 most important variables for the PCA in Figure 2, it becomes apparent that the clustering of maltose; glucose; and rice syrups r01, r02, and r06–r08 are primarily driven by the Raman shifts around 1380–1390 cm^−1^, while the Raman shifts around 1400 cm^−1^ were responsible for the separation of r03 and r04. Therefore, despite the apparent differences between rice syrup spectra, the most important features contributing to their separation on the PCA plot concentrate between 1380 and 1400 cm^−1^. For sugar beet syrups, the dominant sugar was sucrose, at concentrations between 27.33 and 34.63 g/100 g^−1^, followed by fructose and glucose at roughly equal concentrations. For b05, which was used for spiking the honey samples, the concentrations of sucrose, fructose, and glucose were 31.52, 24.74, and 24.37 g/100 g^−1^, respectively.

**Figure 3:**
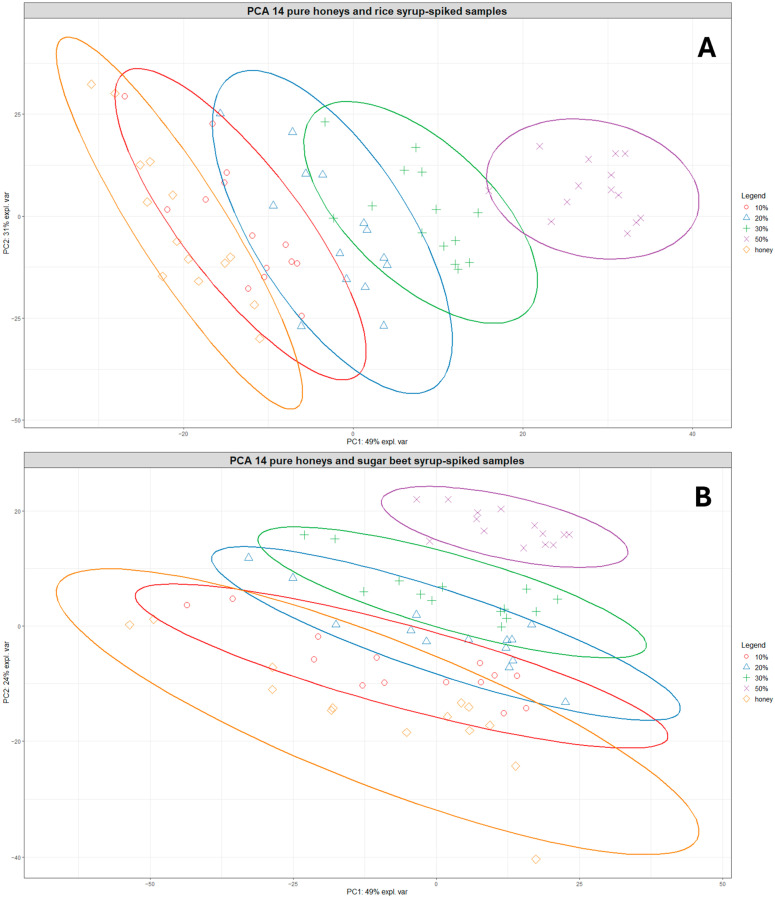
PCA plots showing the clustering of the 14 pure honeys and their corresponding rice syrup-spiked samples at different adulteration levels (**A**) and the clustering of the 14 pure honeys and their corresponding sugar beet syrup-spiked samples at different adulteration levels (**B**). Orange diamonds = pure honeys, red circles = 10% adulteration, blue triangles = 20% adulteration, green crosses = 30% adulteration, and purple exes = 50% adulteration. The ellipses represent 95% confidence ellipses for each group.

Based on the clustering patterns observed in Figure 2, the group of 14 closely clustered pure honeys was selected for developing prediction models for honey adulteration detection. Figure 3A shows the PCA plot of the 14 pure honeys with their corresponding rice-spiked samples. The sample distribution in the PCA plot shows a very clear linear separation according to the levels of adulteration, with the 20–50% adulterated samples positioned separate from the pure honey samples.

A similar pattern was observed for the pure honeys and sugar beet-spiked samples, although there was more overlap between samples belonging to different adulteration level groups (Figure 3B). The samples belonging to the 20–50% adulteration level were also clearly separated from the pure honeys, indicating a limit of detection around 10% for both types of sugar syrups. In addition, Figure S6 shows the clustering of pure and adulterated samples for both rice and sugar beet-spiked samples, which reveals that different levels of the rice-spiked samples are separated over a longer distance along the PC1 axis while sugar beet-spiked samples are much closer together, forming overlapping clusters.

**Figure 4:**
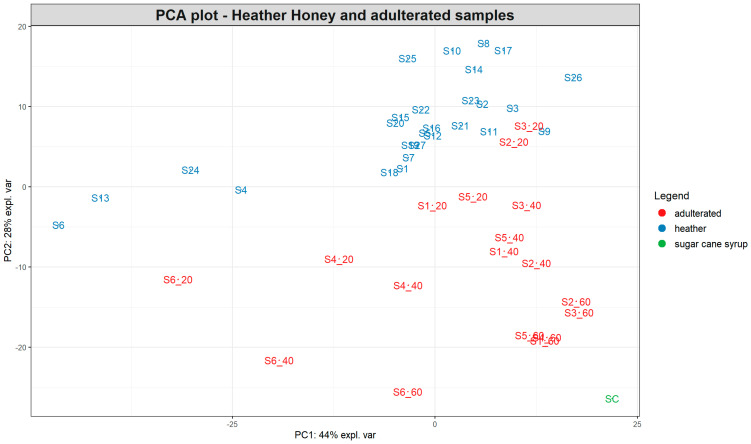
PCA plot based on the SORS spectra of 27 pure heather honeys and 18 heather samples spiked with 20, 40, and 60% partially inverted sugar cane syrup. The number after the underscore represents the percentage of adulteration for each sample. SC = syrup sample.

Finally, the PCA in Figure 4 depicts the clustering of the pure heather honeys and the subsample of sugar cane-spiked samples. In a similar manner to Figure 3, the clustering of samples shows a linear separation between the pure honeys and the sugar cane-adulterated samples stretching both along the PC1 and PC2 axes. The 60% adulterated group is completely separated from the other samples and positioned close to the sugar cane syrup at the bottom right corner of the PCA plot. As shown in Table S2, the sugar composition of partially inverted sugar cane syrup was 21.83, 22.95, and 12.74 mg 100 g^−1^ fructose, glucose, and sucrose, respectively, which was similar to the composition of sugar beet syrups, with the exception of sucrose, which was present in lower concentrations.

Interestingly, most pure heather honeys form a cluster at the upper right side of the PCA plot (Figure 4), with a smaller subgroup occupying the left middle part of the plot separated along the PC1 and PC2 axes. Two of those honeys belong to the “bell heather” group (S4 and S6), while the third bell heather honey (S9) is positioned within the main cluster. The other two honey samples positioned separately (S13 and S24) are ling heather but with a low *Calluna vulgaris* pollen content (7 and 11%, respectively), although this was the case for other ling heather honeys as well within the main cluster. The pollen content of heather honeys varied widely between the different samples, ranging from 3 to 77%.

### 3.2. Classification Model Results

#### 3.2.1. Rice Syrup and Sugar Beet Syrup Adulteration

The performance metrics obtained for each of the algorithms employed for rice and sugar beet syrup adulteration detection are summarised in Table 4. The first two columns show the hard misclassification rates, i.e., the percentage of pure honeys misclassified as adulterated and vice versa, which would be the most serious errors, for all 91 different combinations. The third column summarises the percentage of soft misclassifications, i.e., adulterated samples assigned to the wrong adulteration level. The last column shows the total error rate, i.e., the percentage of misclassified samples out of all the samples tested (*n* = 910).

For the rice syrup classification models, the RF classification algorithm had the best performance overall compared to the other algorithms tested. Only 1.1% of pure honeys were misclassified as adulterated, which corresponds to 2 instances out of the total 182 (91 combinations of 2 pure honeys derived out of 14 pure honeys). In contrast, XGBoost had the highest percentage of pure honeys misclassified as adulterated, at 7.69%, while for PLSA-DA and RF-ordinal, it was 2.2% and 4.4%, respectively.

When considering the hard misclassification rates for the adulterated samples, XGBoost was marginally better, at 1.79%, followed by the RF classification algorithm, at 2.61%. It is also notable that for all algorithms considered, the only adulterated samples misclassified as pure honey belonged to the 10% adulteration level, indicating that the level of detection (LOD) is approximately 10% for all methods.

Regarding the soft misclassifications, both RF algorithms performed better compared to the other algorithms, with 14.15% and 14.97% soft misclassification rates, respectively, exhibiting a higher ability to differentiate between adulteration levels. The PLSDA had similar performance at 16.07% soft misclassifications, while the XGBoost algorithm had the highest misclassification rate at 26.10%. As expected, all samples belonging to the 50% adulteration level were correctly classified by all algorithms.

The total error rate confirmed that the RF classification algorithm performed best, with a 13.63% total error rate, while XGBoost exhibited the highest error rate at 23.85%.

Table 4 also displays the results for the sugar beet classification models, where again, the RF classification model had the best results, with 15.71% total misclassifications, while the XGBoost algorithm had the lowest accuracy out of all, with almost 31% misclassifications. The hard misclassifications for the RF classification model were almost 1% of the pure honey misclassified as adulterated and almost 2% of the adulterated samples misclassified as pure.

The XGBoost results for each sample consisted of the predicted probabilities for each class of adulteration level. Table S1 shows an example of the XGBoost results. The highest probability was chosen as the predicted class of the sample.

Since RF consistently outperformed the other algorithms, it was selected as the algorithm of choice for building a model based on the combined dataset for rice and sugar beet spiked samples. As seen in Table 4, only 1.10% of the pure samples were misclassified as adulterated and 3.64% of the adulterated samples were misclassified as pure. The total misclassifications were 19.23%. In addition, 2.2% of the rice-adulterated samples were misclassified as sugar beet-adulterated and 4.9% of the sugar beet-adulterated samples were misclassified as rice-adulterated.

The spread of total classification accuracy results for the 91 iterations per algorithm and syrup type was further visualised in box plots, as shown in Figure S7. The box plots further demonstrate the superior performance of the RF algorithm, with all the models built with RF (classification and ordinal) having a median accuracy of around 90% and exhibiting left skewness (median > mean), meaning the majority of values were “large”, with a few values towards the low end of the accuracy spectrum. The only exception was the RF classification models for rice syrup adulteration, which had a median around 80% and exhibited right skewness.

#### 3.2.2. Heather Honey Adulteration Detection

As described in Section 3.1, heather honey was among the honey types that exhibited different metabolic fingerprints compared to the 14 honeys which were used to develop the honey authentication models. Heather honey is among the most popular monofloral honeys produced in the UK, and therefore, it would be of interest to develop separate models to capture the unique properties of this special type of honey. For this purpose, we used the data acquired with SORS for the pure and sugar cane syrup-spiked samples to develop preliminary models to showcase the potential of employing SORS for authenticating heather honey. Initially, a binary model was developed using RF algorithm for distinguishing between adulterated and pure heather honey. A total of 100 different models were developed on variations of the training and test data in order to assess the stability of the model performance. The mean accuracy for the binary model was 92% ± 0.01, with 31% of the models having 100% accuracy. Furthermore, the total number of misclassifications per class was extracted, showing that 96.7% of pure honeys were assigned under the correct class, while the success rate for adulterated samples was 86.4%, with some samples in the 20% adulteration group misclassified as pure honey. In addition, we created a model using the RF ordinal algorithm, which correctly predicted the level of adulteration for the samples in the test set. Despite the limited number of samples available, these results showcase that SORS could be used in combination with RF algorithms for developing models similar to the ones presented in Section 2.1 for predicting the level and type of adulteration in heather honeys.

### 3.3. Regression Model Performance

Table 5 shows the mean, max, and min RMSE values acquired for the rice and sugar beet regression models after validating with the test set. RMSE is one of the most widely used measures for the performance of a regression model and the lower the RMSE, the closer the predicted value is to the ground truth. This was also demonstrated by calculating the RMSE values which were similar for both rice and sugar beet, with a mean value of around RMSE = 3.7.

**Figure 5:**
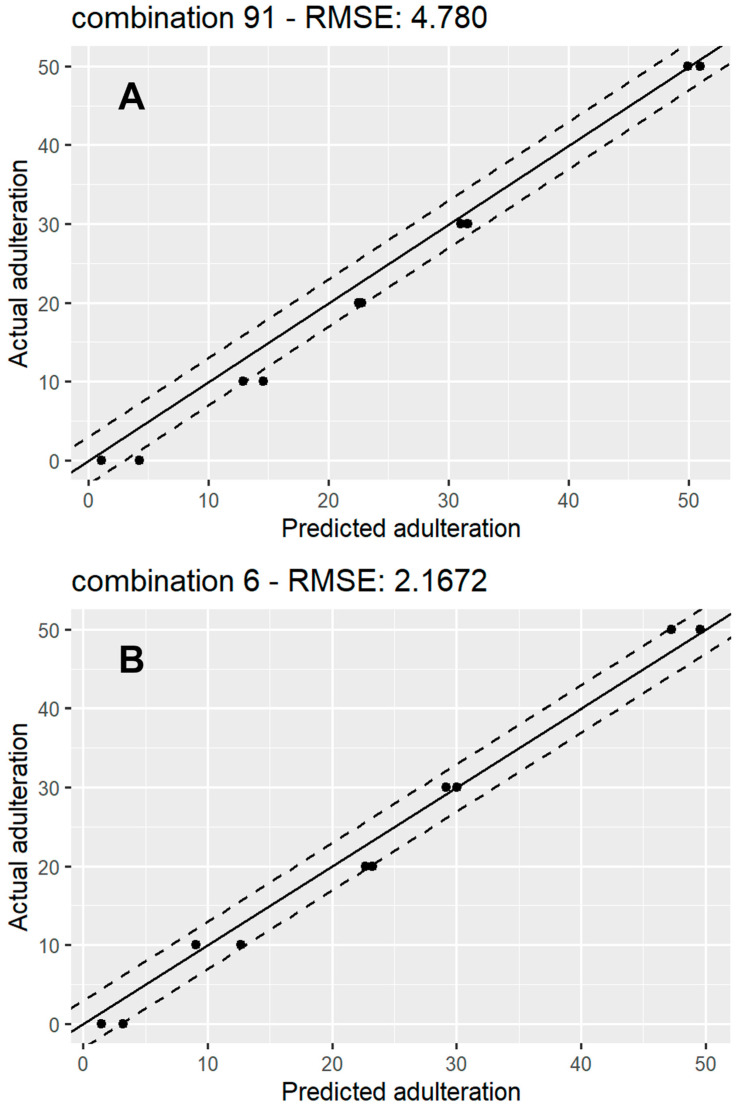
A scatter plot showing actual vs. predicted adulteration percentage for a random RF regression model for rice syrup adulteration (**A**) and sugar beet syrup adulteration (**B**). The solid line represents y = x (perfect agreement between predictions and ground truth), while the top and bottom dashed lines represent y = x + 3 and y = x – 3 respectively.

Figure 5 shows a scatterplot of predicted vs. actual values for 1 of the 91 models for rice syrup adulteration (Figure 5A) and 1 of the sugar beet adulteration models (Figure 5B). A line, x = y, representing perfect agreement between the predictions and the ground truth, has been fitted in each plot, surrounded by y = x ± 3 lines, which represent a 6% mismatch boundary between the predicted values and ground truth, which contained the majority of the predictions for both types of syrups. These plots could also provide a useful indication of bias or large variance indicative of undertraining or overtraining of the models. The absence of these trends from our results provides further proof of the optimised model training process. The spread of the RMSE for the 91 iterations for the rice and sugar beet regression models is displayed through box plots (Figure S8). The plot shows that both have very similar means, with the rice models’ RMSEs having more variation than the sugar beet models.

### 3.4. Variable Importance

**Figure 6:**
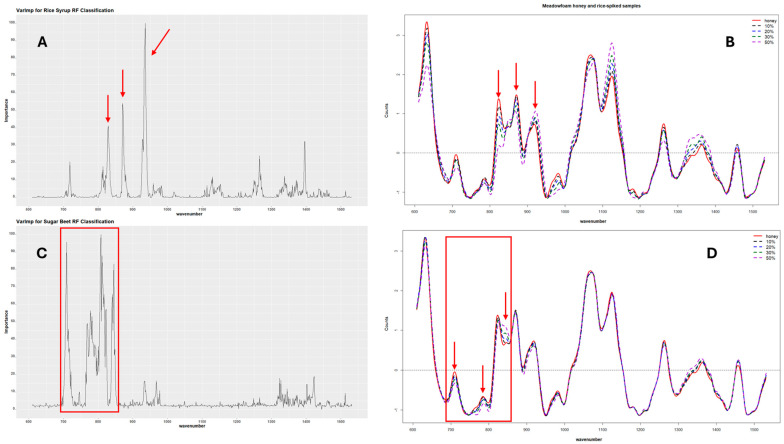
Variable importance for rice and sugar beet classification models. Plot (**A**) shows the variable importance for one of the rice models, and (**B**) shows the SORS spectra for one of the pure honeys and its rice-spiked corresponding samples. Plot (**C**) shows the variable importance for one of the sugar beet models, while (**D**) shows the SORS spectra of one of the pure honeys with its sugar beet-adulterated samples. The arrows and boxes point to the Raman shift areas identified as significant for the model accuracy.

The variable importance for each RF model was also calculated to determine the Raman shifts responsible for the predictive power of the classification and regression models. The results showed general agreement between the Raman bands identified as the most important for the prediction and the areas in the SORS spectra with visible differences between the pure and adulterated honeys. These wavenumbers also correspond to the characteristic peaks identified in the individual sugar spectra, i.e., maltose, glucose, and sucrose, which are the dominant sugars in the sugar syrups. Figure 6 shows an example of the variable importance for one of the rice and sugar beet models developed alongside their corresponding SORS spectra. For rice adulteration, the Raman bands mainly responsible for the predictive power of the classification RF models ranged from 860 cm^−1^ to 890 cm^−1^ and 920 cm^−1^ to 950 cm^−1^, with the peaks at 933–937 cm^−1^ having the highest contribution. For the sugar beet models, the highest peaks were in the range from 700 cm^−1^ to 850 cm^−1^ with the wavenumbers at 808, 711, 839, 718, and 715 cm^−1^ showing the highest contribution in order of importance. Figure S9 shows that the most important variables for the combined model were located between 700 cm^−1^ and 950 cm^−1^, with the highest peak at ~935 cm^−1^, which corresponds to a combination of the important variables for the individual models.

The variables identified as most important for the RF regression models were also similar to the classification models (Figure S10). The most important variables for the rice adulteration model were the Raman shifts at 873, 870, and 939 cm^−1^, in order of importance, while for the sugar beet adulteration models, the most important variables in order of importance were the Raman shifts at 781, 775, 708, 808, and 839 cm^−1^.

Finally, the ordinal RF model for heather honey adulteration with sugar cane syrup revealed that the area between 1047 and 1126 cm^−1^ was important for the predictive accuracy of the model, with 1060 cm^−1^ having 100% relative importance compared to the other variables. Another area of importance was the Raman shift at around 838 cm^−1^, which had the most significant contribution to the predictive capacity of the model (55% relative contribution).

## 4. Discussion

In this study, we successfully employed through-container SORS measurements coupled with machine learning predictive modelling to achieve non-invasive detection of exogenous sugar adulteration in UK honeys. Different levels of adulteration, between 10 and 60%, were employed to train various machine learning algorithms, including PLSDA, RF, and XGBoost. The syrups used for adulteration were rice, sugar beet, and sugar cane. Among them, rice and sugar beet syrups derive from C3 plants, which are not easily detected with existing methods. Thus, it is important to develop methods targeting these sources of exogenous sugars.

The results revealed that SORS successfully differentiated honeys from plant-based syrups according to their spectral fingerprint. Moreover, the majority of honey samples had a similar biochemical profile, mainly characterised by the presence of fructose and glucose, with the exception of three honey types (heather, ivy, and buckwheat), which were positioned away from the main cluster of pure honeys in the PCA plot (Figure 2). This difference could be attributed to biochemical differences, as indicated by their colour differences and characteristic SORS signals. Indeed, the 14 honeys in the main cluster were all white—light—amber-coloured honeys, while the other 3 honeys (heather, ivy, and buckwheat) ranged between amber and a dark amber colour, resulting in higher intensity SORS signals as well as strong fluorescence interference (Figure S1). Heather honey is known to have distinct physicochemical properties among honeys, such as higher moisture contents and sucrose/glucose ratios. In addition, it is rich in bioactive compounds such as phenolics [30,31], which possibly contribute to the high signal intensity and fluorescence interference. The same trend was observed for the 27 heather honeys from Year 1, which despite the high variability in *Calluna vulgaris* pollen content ranging between 3 and 77%, exhibited similar metabolic fingerprints, as shown by their SORS signal and PCA plot (Figure 4), indicating it would be possible to build a single model for authenticating this type of honey. It is notable that while honeys from other floral sources are considered monofloral when ≥45% of the pollen belongs to a single species, heather pollen is considered an underrepresented pollen, and as a result, heather honeys can be characterised as monofloral at a ≥20% *Calluna vulgaris* [32] pollen content as long as they exhibit the physicochemical characteristics of heather honey.

### RF Algorithm Successfully Discriminates Pure Honey

Classification models built with the RF classification algorithm consistently outperformed alternative algorithms, exhibiting misclassification rates of 1.1% for pure honey misclassified as adulterated and 2.0% to 2.6% for adulterated honey misclassified as pure (hard misclassifications). The total misclassification rates ranged from 13.6% to 15.7%. Conversely, XGBoost displayed higher misclassification rates, ranging from 7.7% to 9.9% for pure honey and an overall misclassification rate of approximately 23.9% to 31.0%. The utilisation of XGBoost was motivated by its ability to provide probability-based results for each adulteration class (refer to the example in Table S1). However, its performance was less than that of RF despite both being decision tree-based ensemble algorithms. A possible cause for this could be the slow training process of XGBoost due to the many hyperparameters involved, which can lead to overfitting if not adequately optimised. Another defining difference was the fact that XGBoost employs regularisation to avoid weight overinflation to reduce the risk of overfitting. Indeed, the final XGBoost models following the training and optimisation process only contained 79 features out of the 670 original features. RF, in contrast, builds hundreds of trees using different combinations of training samples and features for each of the trees, therefore minimising the risk of overfitting without eliminating features. In fact, our efforts to reduce the feature space prior to performing RF model training were less successful compared to employing the whole feature space, as described in Section 2.6, possibly indicating the presence of complex interactions between features in the SORS spectra. Other studies on honey authenticity have compared the performance of several machine learning algorithms, with both RF and XGBoost reaching similar performances. XGBoost exhibited 90% accuracy in discriminating between monofloral Spanish honeys of different botanical origins based on honey physicochemical parameters as compared to 83% accuracy for RF [33]. In another study employing hyperspectral imaging to study adulteration detection in Pakistani honeys, RF had superior performance in identifying both the botanical origin and adulteration level, reaching a maximum prediction accuracy of 99.69%, while XGBoost attained similar levels of performance [34].

In addition to its increased performance, another advantage of using RF was that it provides a variable importance matrix ranking the features in order of their impact in the model’s prediction accuracy. The wavenumbers of highest importance for the different rice syrup classification models ranged from 860 to 890 cm^−1^ and 920 to 950 cm^−1^. These wavenumbers correspond to two peaks characteristic of maltose, which were also present in rice syrup, showing that the algorithm correctly identified maltose as the sugar responsible for differentiating between natural honey and honey adulterated with rice syrup. Shuhan et al. (2022) [12] have also identified two Raman peaks at 865 and 915 cm^−1^ among the most important features for the identification of the maltose syrup adulteration of native Suichang Chinese honey, which indicate the presence of C-H and C-H and C-OH bond bending vibrations. The regression models for rice syrup adulteration also showed that the wavenumbers around 865 cm^−1^ contributed more than 50% to the prediction of adulteration level (Figure S10).

The bands responsible for the prediction accuracy of the RF sugar beet models (both classification and regression), ranged from 700 to 850 cm^−1^. The band around 706 cm^−1^ has been previously associated with C-O stretching and C-C-C-O and O-C-O bending vibrations of glucose [35]. The area around 840 cm^−1^ also coincided with a dominant peak in the sucrose SORS spectrum—attributed to the ν(C-C) stretches of glucose—and is in agreement with previous observations by Mosca et al. (2023) [22]. The SORS results were further validated by HPLC analysis, which revealed distinctive sugar profiles for rice and sugar beet syrups, with the rice syrup predominantly consisting of glucose and maltose, while the sugar beet and sugar cane syrups predominantly containing sucrose, glucose, and fructose. Furthermore, the main Raman bands around the 840 and 1060 cm^−1^ Raman shifts associated with sugar cane adulteration can be attributed to the presence of sugars and possibly proteins. More specifically, the band at ~840 cm^−1^ is attributed to the presence of glucose and the torsion motion of the CH_2_ groups (as reviewed by Wiercigroch et al. (2017) [36]), while the 1048 cm^−1^ band is thought to have originated from the ν(C-O) vibration of the glucose ring. The 1057 cm^−1^ shift could be caused by a major contribution by the bending vibration of C(1)-H and COH in carbohydrates and a minor contribution by the vibration of C-N bond in proteins and amino acids [10].

RF was further used to develop a classification model, with the aim of discerning the type of sugar syrup used for adulteration, as well as predicting the level of adulteration. This model also exhibited a high success rate, with only 1.10% of pure samples misclassified as adulterated and 3.64% of adulterated samples misclassified as pure. In addition, it exhibited superior performance in distinguishing the type of adulterant, with up to a 4.90% misclassification rate. The fact that this model was able to successfully identify the type of adulteration was of high importance, as it showcases that SORS accurately captures differences in chemical composition between different syrup types and honeys. As demonstrated in Figure S11, the Raman bands with the highest importance for the combined model ranged between 700 and 950 cm^−1^, which encompasses the most important variables previously identified for the individual rice and sugar beet classification models. In addition, a band around 1127 cm^−1^ has previously been suggested to arise from a combination of the stretching vibration of the C-O bond (major) and the vibration of the C-N bond of protein and amino acids (minor), while the band around 1264 cm^−1^ has been associated with the vibration of C(6)-OH and C(1)-OH bonds [37].

While previous studies have shown promising results of employing Raman spectroscopy to identify honey adulteration with sugar syrups, they are not able to specify the type of sugar syrup used for adulteration [22], or in some cases, they show poor performance compared to individual models [37]. In contrast, the SORS methodology exhibited a high discriminatory capacity with potential applications in testing unknown honey samples in non-controlled commercial settings where the type of adulterant would be unknown.

Similar to the results observed for classification purposes, the regression models employing RF regression methods demonstrated very low RMSE values, around 3.7, for both rice and sugar beet syrups, with SDs of 1.67 and 1.09, respectively. These results demonstrate the efficacy of these models in predicting adulteration levels with high accuracy, adding a valuable quantitative dimension to the study.

Finally, as shown in the present study, the majority of pure honey types in the UK had a similar metabolic fingerprint, which allowed us to group them together in a single model. Additionally, individual models can be constructed in the future to characterise more diverse types of honey with unique fingerprints, such as heather, ivy, and buckwheat. The results we obtained for heather honeys from the UK support this hypothesis.

This study emphasises the potential of SORS and machine learning as a cost-effective and rapid method for honey authentication. The deployment of SORS in the field and a through-container analysis offers practical applications for adulteration detection and quality monitoring in the honey supply chain. Future research is recommended to refine the models, reduce misclassifications, and broaden the study to encompass a diverse range of commercial honey types.

In addition, SORS exhibited quantitative potential, showing that the proposed new methodology could be used both for a qualitative analysis to detect the presence/absence of adulterant and to quantify the percentage of adulteration. More work on blended honeys would be required if this approach is to be extended to imported honeys, which constitute the majority of honey available in the UK market [2]. This would involve constructing reference databases with commercially relevant samples.

## 5. Conclusions

In conclusion, the results of this study demonstrate the potential of SORS coupled with multivariate analysis methods for the authentication of UK honey and adulteration detection at a ~10% LOD. The portability and ease of use of SORS makes it an attractive screening tool for testing honey, enhancing traceability and quality control. The proof of concept demonstrated for UK honeys could be extended to other regions and types of honey in the future. A prerequisite for this would be the construction of comprehensive databases with a large selection of different types of honey sourced at various stages of the supply chain, including harvest, blending, heat treatment, and transport until the final retail destinations, as well as different types of sugar syrups, which would be used to build representative predictive models able to generalise well to future samples. A SORS-based screening method could serve as a quick tool to differentiate between pure and “suspicious” samples, which would then undergo further tests before a decision is reached based on the weight of evidence. This approach could improve the capability of regulators and industry to protect consumer and verify supply chains.

## Figures and Tables

**Table 1:** Heather honeys collected in Year 1, and the percentage of ling heather (*Calluna vulgaris*) pollen detected using the pollen count method.

Sample Code	Label	Area	*Calluna vulgaris* Pollen (%)
S1	Sample1	Scotland	52
S2	Sample2	Shropshire	77
S3	Sample3	Scotland	19
S4	Sample4	Dorset	NA *
S5	Sample5	Scotland	55
S6	Sample6	Dorset	NA *
S7	Sample7	Wales	10
S8	Sample8	Yorkshire	37
S9	Sample9	Yorkshire	NA *
S10	Sample10	Devon	4
S11	Sample11	Devon	9
S12	Sample12	Scotland	11
S13	Sample13	Scotland	7
S14	Sample14	Wales	20
S15	Sample15	Shropshire	12
S16	Sample16	Shropshire	43
S17	Sample17	Yorkshire	20
S18	Sample18	Wales	3
S19	Sample19	Scotland	13
S20	Sample20	Yorkshire	12
S21	Sample21	Scotland	10
S22	Sample22	Wales	70
S23	Sample23	Shropshire	16
S24	Sample24	Sheffield	11
S25	Sample25	Scotland	69
S26	Sample26	Derbyshire	4
S27	Sample27	Scotland	37

* NA = not available (honey named as predominantly bell heather by producers).

**Table 2:** Description, season, floral sources, and UK origin of the honey samples collected in Year 2.

Sample Number	Honey Description	Season	Floral Sources *	UK Region
H1	woodland	summer	Woodland trees and nearby flowers, including lime (*Tilia*), horse chestnut (*Aesculus hippocastanum),* and sweet chestnut (*Castanea sativa*).	Yorkshire
H2	sycamore	spring	predominantly sycamore with a bit of hawthorn and bean	Yorkshire
H3	phacelia	spring	*Phacelia tanacetifolia*	Yorkshire
H4	ivy	autumn	*Hedera helix*	Yorkshire
H5	Himalayan balsam	autumn	*Impatiens glandulifera*	Yorkshire
H6	spring set	spring	multifloral	Yorkshire
H7	borage	summer	*Borago officinalis*	Warwickshire
H8	buckwheat	autumn	*Fagopyrum esculentum*	Yorkshire
H9	meadowfoam	summer	*Limnanthes alba*	Warwickshire
H10	sea lavender	summer	*Limonium vulgare*	Norfolk
H11	heather	autumn	*Calluna vulgaris*	Exmoor
H12	echium	summer	*Echium plantagineum*	Warwickshire
H13	field and forest	blend	a heather blend multifloral honey with moor, woodland, and wild pasture flower honey	Yorkshire
H14	hedgerow	blend	hedgerows, meadows, and farmland	Norfolk
H15	English blossom	spring and summer	blend of blossoms from spring and summer	Yorkshire
H16	apple blossom	spring	*Malus domestica*	Norfolk
H17	wildflower	summer	mixture of wildflowers including Bluebell, Cowslip, Gorse, Orchids, Honeysuckle, Meadowsweet, Lime, Rosebay willow herb, and St John’s Wort	Warwickshire

* Indicates floral sources of a sample as obtained from a honey producer.

**Table 3:** Sugar syrup product information.

ID	Product	Details *
r01	rice syrup	Spanish rice molasses (organically grown rice molasses), 460 g
r02	rice syrup	Spanish rice syrup (water, organic rice (35%)), 400 g
r03	rice syrup	Korean rice syrup (rice starch 100%), 700 g
r04	rice syrup	Korean rice syrup (rice 100%), 700 g
r06	rice syrup	German rice syrup (organic rice flour (82%), water, non-EU), 1.4 kg
r07	rice syrup	UK organic rice syrup, non-EU agriculture (93% rice, 7% water), 250 g
r08	rice syrup	UK organic rice syrup, EU/non-EU agriculture (rice (93%), water), 350 g
b01	sugar beet molasses	German sugar beet syrup (sugar beet 100%—organic), 1 L
b05	sugar beet syrup	Swedish light syrup (syrup from Swedish sugar beets, salt), 750
b06	sugar beet syrup	UK golden syrup (partially inverted sugar syrup), 680 g
b07	mix of sugar beet syrup and sugar cane syrup	Swedish dark syrup (syrup from Swedish sugar beets, cane sugar syrup, and salt), 750 g
Sugar cane	refiner’s syrup	UK golden syrup (partially inverted refiners’ syrup), 325 g

* Details obtained from the product packaging, including country of company of purchase.

**Table 4:** Results of the classification models for the rice syrup-adulterated and sugar beet syrup-adulterated samples, as well as the results for the RF classification model for the adulterated samples combined (rice and sugar beet).

Models	Pure Honey Misclassified as Adulterated	Adulterated Misclassified as Pure Honey	Soft Misclassifications	Total Misclassifications
		Rice Syrup Models		
PLSDA	2.20%	8.79%	16.07%	20.33%
RF_Classification	1.10%	2.61%	14.15%	13.63%
RF_Ordinal	4.40%	5.63%	14.97%	17.36%
XGBoost	7.69%	1.79%	26.10%	23.85%
		Sugar Beet Syrup Models		
PLSDA	1.10%	1.92%	19.51%	17.36%
RF_Classification	1.10%	2.06%	17.31%	15.71%
RF_Ordinal	1.10%	1.92%	17.72%	15.93%
XGBoost	9.89%	4.53%	31.73%	30.99%
		Rice and Sugar Beet Combined Model		
RF_Classification	1.10%	3.64%	17.86%	19.23%

**Table 5:** Summary statistics of the RMSE results obtained for the rice and sugar beet adulteration regression models with RF based on the test set validation. SD = standard deviation.

RF Regression	Mean RMSE	SD RMSE	Mean R^2^	SD R^2^
Rice Syrup	3.69	1.67	0.96	0.0049
Sugar Beet Syrup	3.67	1.09	0.97	0.0035

## Data Availability

Data supporting this study are included within the article and Supporting Materials, further inquiries can be directed to the corresponding author. The underlying data can be accessed at CERES (https://dspace.lib.cranfield.ac.uk/handle/1826/22688, accessed on 31 March 2023).

## Supplementary Materials

The following supporting information can be downloaded at https://www.mdpi.com/article/10.3390/foods13152425/s1, Figure S1: Honey samples (left to right: 1: woodland, 2: sycamore, 3: phacelia, 4: ivy, 5: himalayan balsam, 6: spring set, 7: borage, 8: buckwheat, 9: meadowfoam, 10: sea lavender, 11: heather, 12: echium, 13: field & forest, 14: hedgerow, 15: blossom, 16: apple blossom, 17: wildflower); Figure S2: Syrup samples used for SORS (left to right rice syrup r01-r04 and r06-r08, and sugar beet syrup b05, b01, b06 and b07). Figure S3. An example of the raw SORS spectrum for the three technical replicates acquired for one of the honey samples in Year2 (woodland honey); Figure S4: Schema describing the selection of the training and test sets in Year2. The dataset consisted of 14 pure honeys and their rice-spiked and sugar beet-spiked samples. In each iteration, 12 honeys and their spiked samples were chosen as the training set, and the remaining 2 honeys and their spiked samples were chosen as the test set. This process was done separately for the rice and sugar beet models; Figure S5: Pre-processed SORS spectra of rice syrups, maltose and glucose aqueous solutions 50% *w*/*v* (A). PCA biplot of the SORS spectra acquired for honeys, sugar syrups and sugar aqueous solutions. The arrows represent the 150 most influential variables in the SORS spectra for the PCA; Figure S6: PCA showing the 14 pure honeys with their rice-spiked and sugar beet-spiked samples; Figure S7: Boxplots showing accuracy dispersion for all the 91 classification models created for rice (red boxplots) and sugar beet (blue boxplots) syrups per algorithm (plsda = PLS-DA, rf_classification = RF classification, rf_ordinal = RF ordinal, xgboost = XGBoost). The dots within the boxes represent the mean, while the horizontal line represents the median; Figure S8: Box plots showing the RMSE spread for all 91 combinations for the rice (red) and sugar beet (blue) models. The dots in the boxes represent the mean, while the horizontal line represents the median; Figure S9: Variable importance for one of the rice and sugar beet combined classification models; Figure S10: Variable importance for rice and sugar beet regression models. Plot A shows the variable importance for one of the rice models, and B shows the variable importance for one of the sugar beet adulteration models. The dashed horizontal line divides the Raman shifts having relative variable importance ≥ 50% from the Raman shifts with variable importance ≤ 50%; Figure S11: SNV Normalised spectra of three pure heather honeys and a partially inverted sugar cane syrup (orange line); Table S1: Example of a confusion matrix for one of the XGBoost classification models for rice adulteration showing results in the form of probabilities for each class; Table S2: Sugar concentrations (g/100g-1) in rice syrup, sugar beet and partially inverted sugar cane syrup (SC) samples.
