# Supplementary material for: Application of Spatial Offset Raman Spectroscopy (SORS) and Machine Learning for Sugar Syrup Adulteration Detection in UK Honey
Source: Foods. 2024 Jul 31;13(15):2425. doi: 10.3390/foods13152425 (PMC11312281; doi:10.3390/foods13152425)
Supplement: Supplementary file 1 [file foods-13-02425-s001.zip › foods-3110388-supplementary.pdf]

foods

Article

Application of Spatial Offset Raman Spectroscopy (SORS) and Machine Learning for Sugar Syrup Adulteration Detection in UK Honey

Mennatullah Shehata [1], Sophie Dodd [1], Sara Mosca [2], Pavel Matousek [2], Bhavna Parmar [3], Zoltan Kevei [1] and Maria Anastasiadi [1,*]

[1] Centre for Soil, Agrifood and Biosciences, Cranfield University, College Road, Cranfield, Bedford MK43 0AL, UK
[2] Central Laser Facility, Research Complex at Harwell, STFC Rutherford Appleton Laboratory, UKRI Harwell Campus, Didcot OX11 0QX, UK
[3] Food Standards Agency, Clive House, 70 Petty France, Westminster, London SW1H 9EX, UK
* Correspondence: m.anastasiadi@cranfield.ac.uk

Supplementary Information

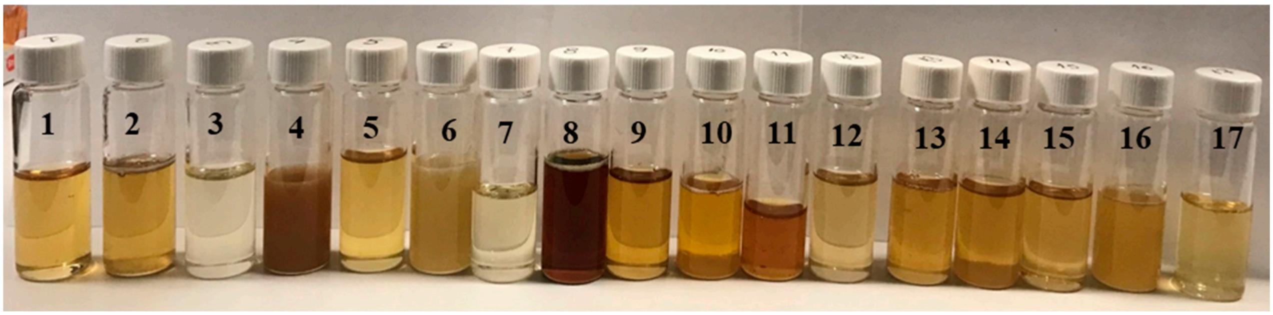

Figure S1. Honey samples (left to right: 1: woodland, 2: sycamore, 3: phacelia, 4: ivy, 5: himalayan balsam, 6: spring set, 7: borage, 8: buckwheat, 9: meadowfoam, 10: sea lavender, 11: heather, 12: echium, 13: field & forest, 14: hedgerow, 15: blossom, 16: apple blossom, 17: wildflower).

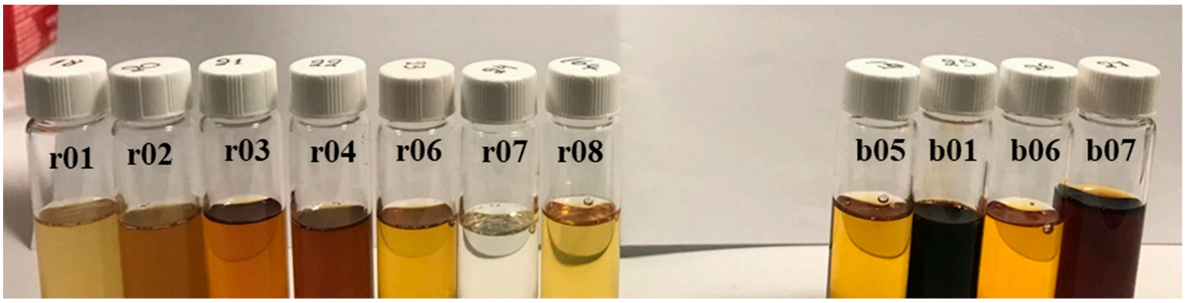

Figure S2: Syrup samples used for SORS (left to right rice syrup r01-r04 and r06-r08, and sugar beet syrup b05, b01, b06 and b07).

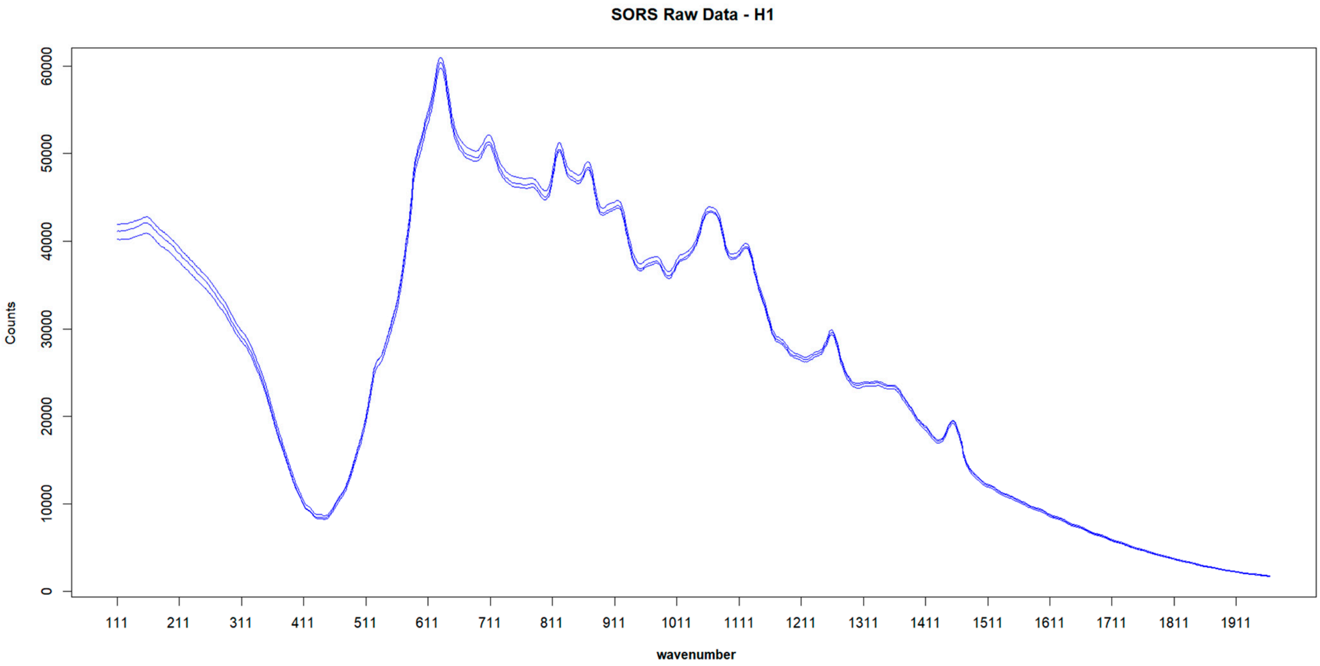

Figure S3. An example of the raw SORS spectrum for the three technical replicates acquired for one of the honey samples in Year2 (woodland honey).

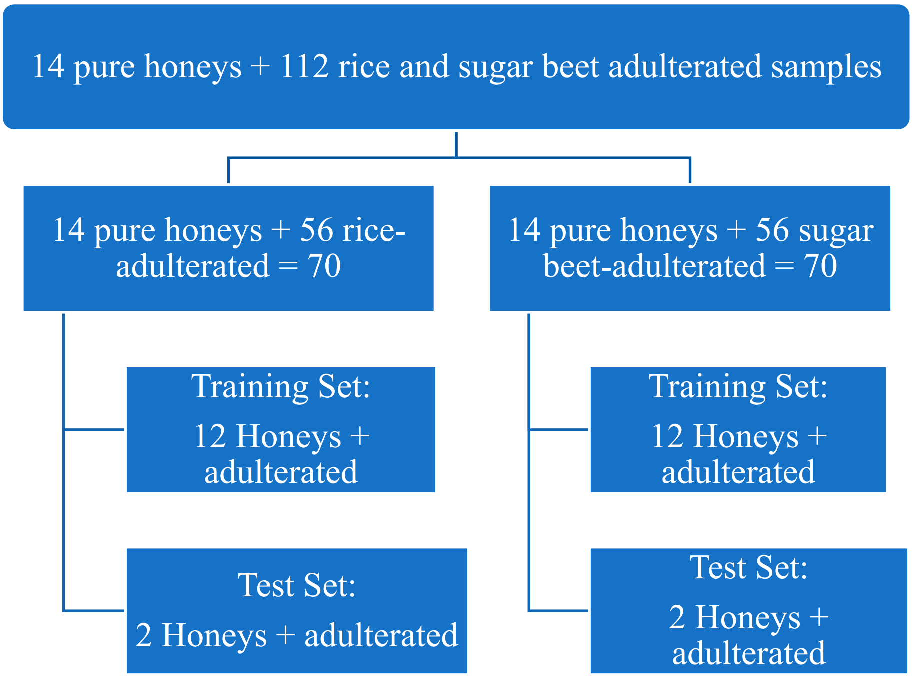

Figure S4. Schema describing the selection of the training and test sets in Year2. The dataset consisted of 14 pure honeys and their rice-spiked and sugar beet-spiked samples. In each iteration, 12 honeys and their spiked samples were chosen as the training set, and the remaining 2 honeys and their spiked samples were chosen as the test set. This process was done separately for the rice and sugar beet models.

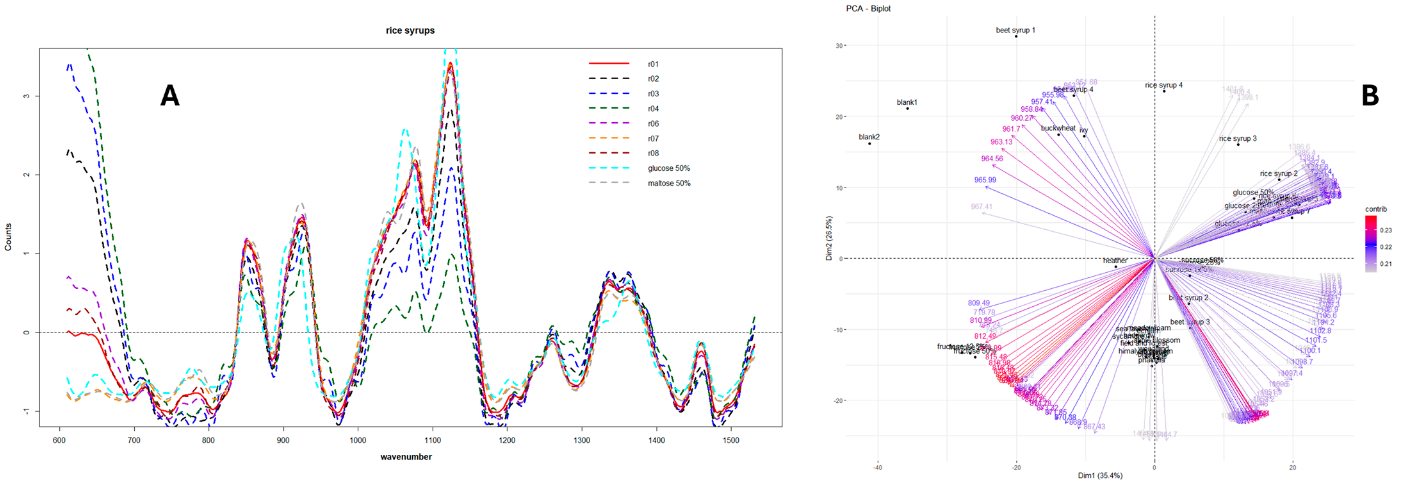

Figure S5. Pre-processed SORS spectra of rice syrups, maltose and glucose aqueous solutions 50% w/v (A). PCA biplot of the SORS spectra acquired for honeys, sugar syrups and sugar aqueous solutions. The arrows represent the 150 most influential variables in the SORS spectra for the PCA.

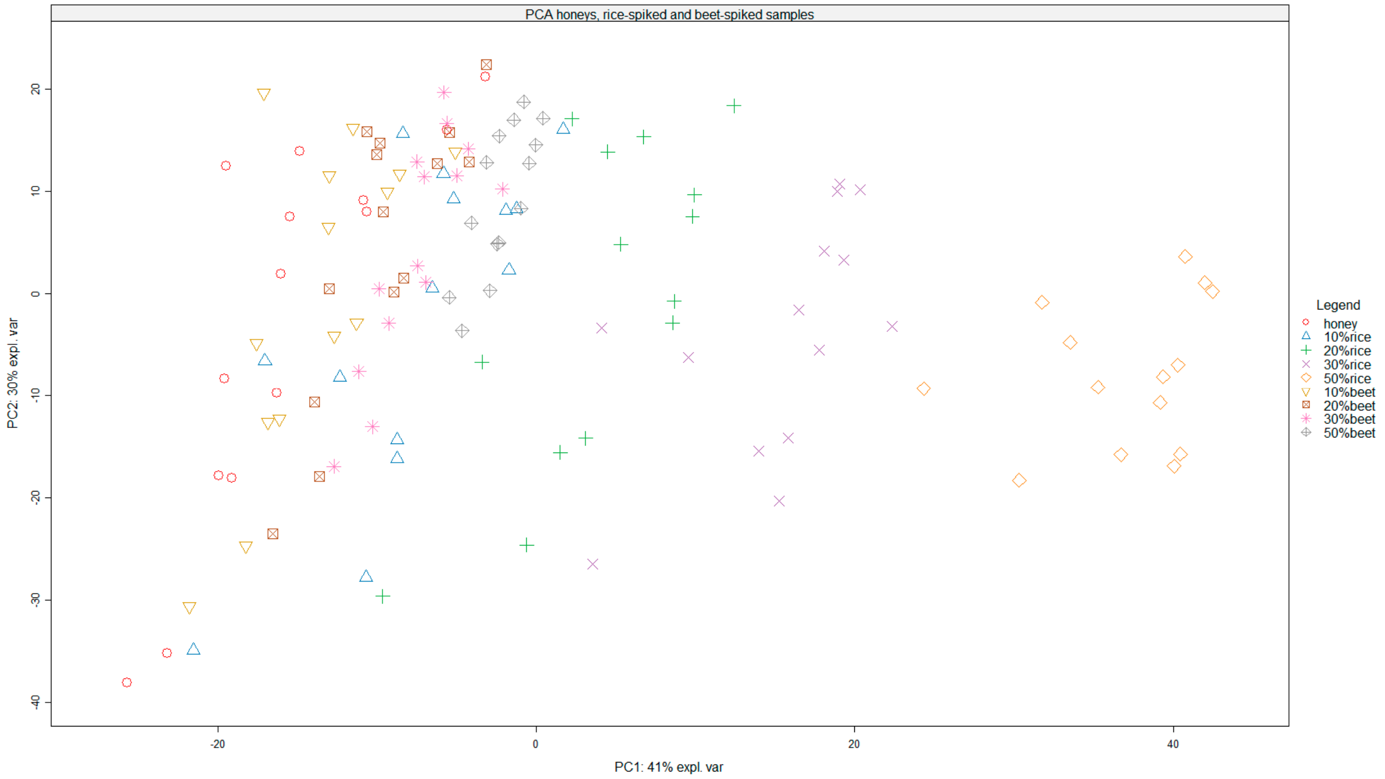

Figure S6. PCA showing the 14 pure honeys with their corresponding rice-spiked and sugar beet syrup-spiked samples. The different symbols and colours indicate different types of samples and adulteration levels as explained in the figure legend.

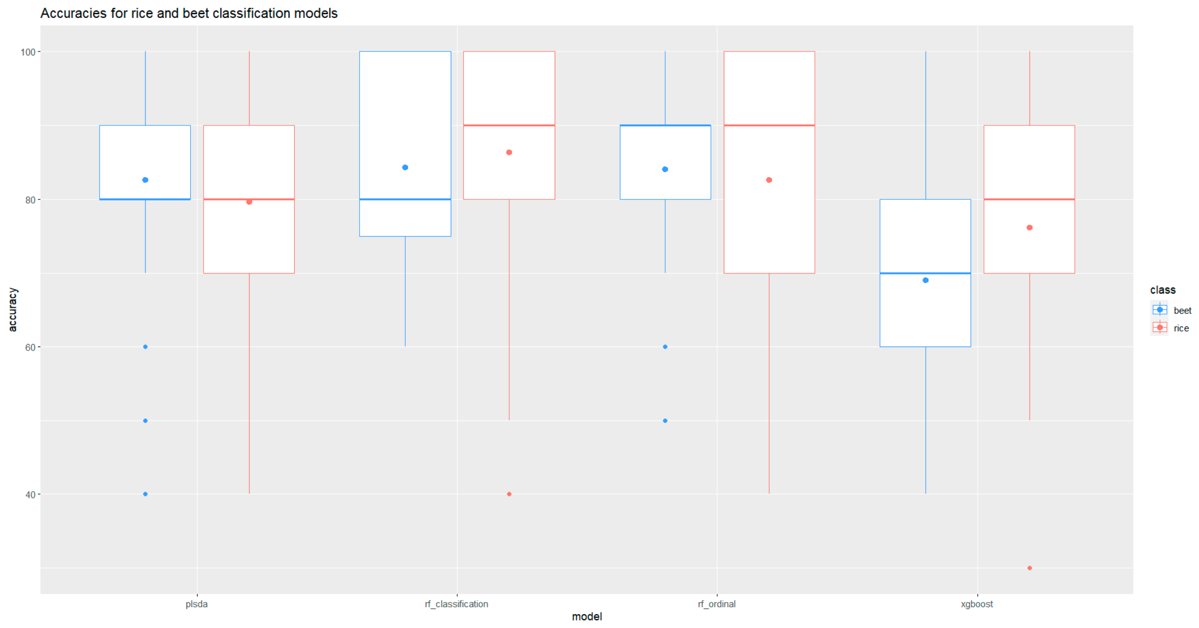

Figure S7. Boxplots showing accuracy dispersion for all the 91 classification models created for rice (red boxplots) and sugar beet (blue boxplots) syrups per algorithm (plsda = PLS-DA, rf_classification = RF classification, rf_ordinal = RF ordinal, xgboost = XGBoost). The dots within the boxes represent the mean, while the horizontal line represents the median.

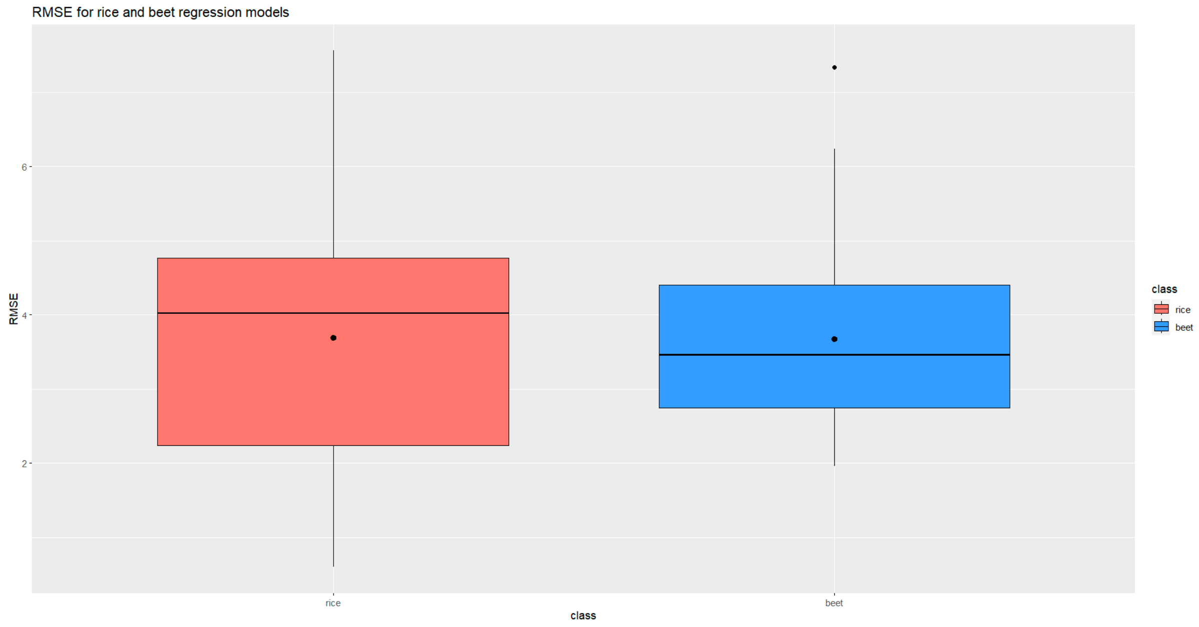

Figure S8. Box plots showing the RMSE spread for all 91 combinations for the rice (red) and sugar beet (blue) models. The dots in the boxes represent the mean, while the horizontal line represents the median.

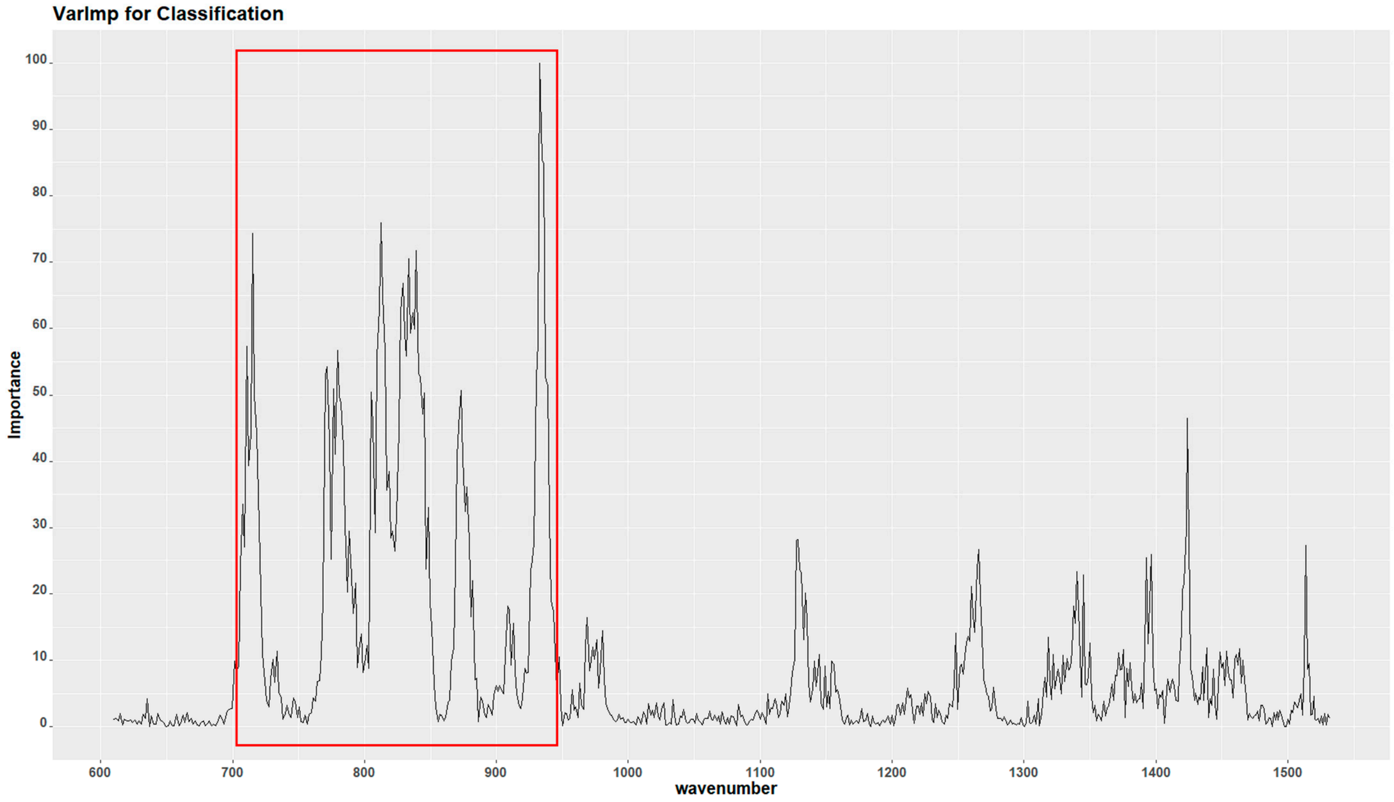

Figure S9: Variable importance for one of the rice and sugar beet combined classification models.

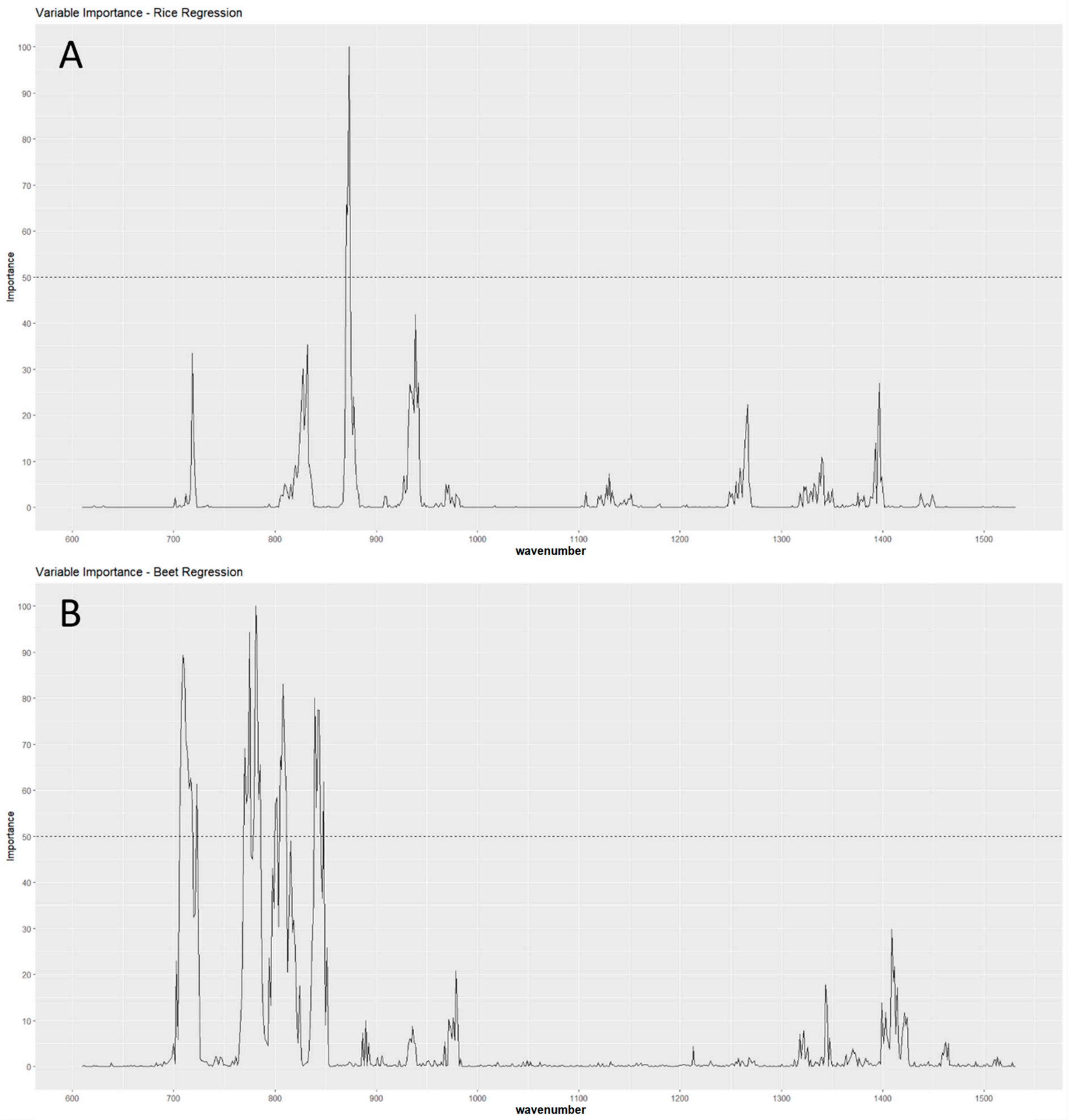

Figure S10. Variable importance for rice and sugar beet regression models. Plot A shows the variable importance for one of the rice models, and B shows the variable importance for one of the sugar beet adulteration models. The dashed horizontal line divides the Raman shifts having relative variable importance ≥ 50% from the Raman shifts with variable importance ≤ 50%.

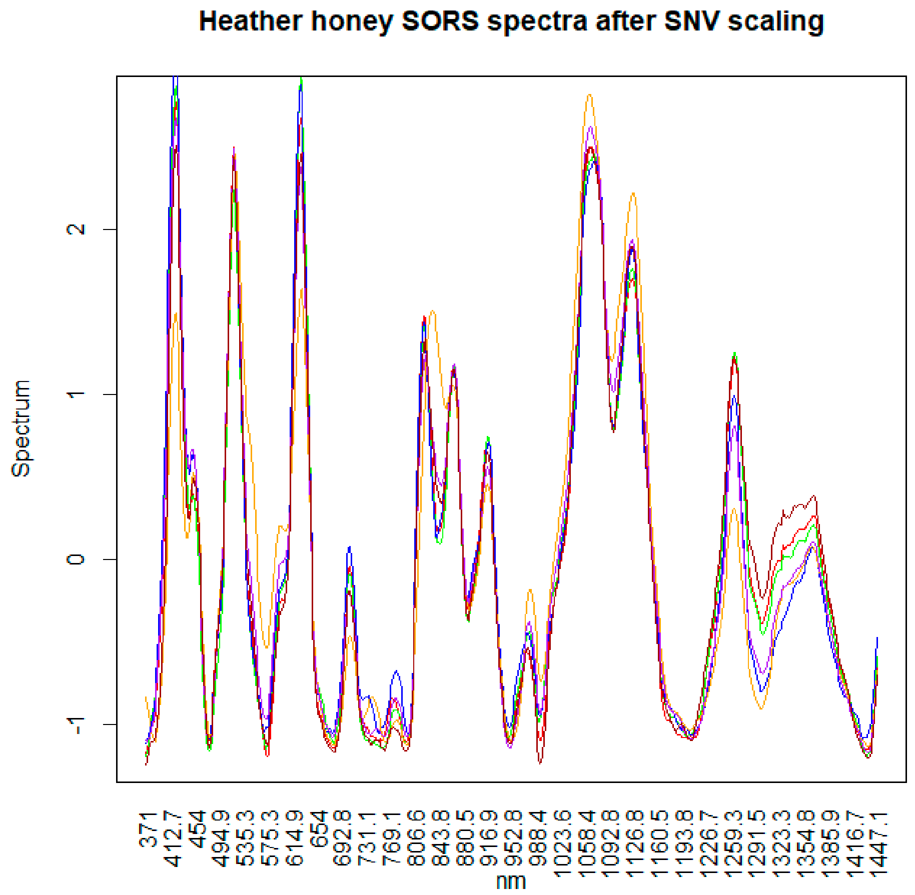

Figure S11. SNV Normalised spectra of three pure heather honeys and a partially inverted sugar cane syrup (orange line).

Table S1: Example of a confusion matrix for one of the XGBoost classification models for rice adulteration showing results in the form of probabilities for each class.

Reference	honey	10%	20%	30%	50%
**honey**	92.20%	5.49%	1.13%	0.62%	0.56%
**honey**	97.72%	0.94%	0.29%	0.46%	0.59%
**10%**	1.78%	72.12%	21.61%	2.74%	1.75%
**20%**	6.27%	4.71%	54.44%	28.45%	6.14%
**30%**	4.08%	2.54%	22.01%	67.37%	4.00%
**50%**	0.72%	0.41%	0.97%	1.55%	96.35%
**10%**	5.45%	76.72%	7.56%	4.93%	5.34%
**20%**	9.71%	22.83%	44.13%	13.82%	9.51%
**30%**	4.53%	2.82%	21.45%	66.77%	4.44%
**50%**	0.72%	0.41%	0.50%	1.86%	96.51%

Table S2. Sugar concentrations (g/100g^{-1}) in rice syrup, sugar beet and partially inverted sugar cane syrup (SC) samples.

Sample	Fructose (g/100g^{-1})	Glucose (g/100g^{-1})	Sucrose (g/100g^{-1})	Maltose (g/100g^{-1})
r01	0.00	22.49	0.22	31.90
r02	0.00	23.31	0.25	28.57
r03	1.68	17.34	0.85	42.59
r04	1.77	9.54	1.51	28.94
r06	0.00	23.01	0.32	36.70
r07	1.79	26.90	0.62	37.14
r08	3.58	28.11	1.49	33.10
b01	19.03	16.40	27.33	0.21
b05	24.74	24.37	31.52	0.27
b06	23.88	26.97	34.63	0.40
b07	24.13	23.67	31.64	0.28
SC	21.83	22.95	12.74	0.00
